# Modified ferriman-gallwey score and hirsutism among normal healthy female population

**DOI:** 10.12669/pjms.40.3.8138

**Published:** 2024

**Authors:** Samina Bibi, Suleman Elahi Malik, Saima Zeb, Javeria Javed

**Affiliations:** 1 Dr. Samina Bibi, MBBS, FCPS. Department of Diabetes, Endocrinology and Metabolic Diseases, MTI Hayatabad Medical Complex, Peshawar, Pakistan; 2 Dr. Suleman Elahi Malik, MBBS, FCPS Medicine, FCPS Endocrinology, Endocrinology Division, Department of Medical Specialties, MTI Khyber Teaching Hospital, Peshawar, Pakistan; 3 Dr. Saima Zeb, MBBS, FCPS, MRCP. Department of Diabetes, Endocrinology and Metabolic Diseases, MTI Hayatabad Medical Complex, Peshawar, Pakistan; 4 Dr. Javeria Javed, MBBS. Department of Medicine, MTI Khyber Teaching Hospital, Peshawar, Pakistan

**Keywords:** Modified Ferriman-Gallwey Score, Hirsutism, Normal Healthy Female Population

## Abstract

**Objective:**

This study aimed to investigate the prevalence of Hirsutism by using the mFG score and to identify the mean mFG score among the normal healthy female population of Peshawar.

**Methods:**

A cross-sectional study was conducted among 448 normal healthy married women aged between 20 to 40 years from 14^th^ April 2022 to 13^th^ October 2022 at Hayatabad Medical Complex in Peshawar. The mFG score was used to evaluate Hirsutism, a score of eight or above was regarded as indicative of Hirsutism.

**Results:**

The mean modified Ferriman-Gallwey (mFG) score was 8.89 ± 4.33. 255 (56.9%) of the individuals had a mFG score of more than 8. These people showed mild hirsutism in 52.0% of cases, moderate hirsutism in 4.5% of cases, and severe hirsutism in 0.4% of cases. It was observed that the lower abdomen and thigh region had the highest prevalence of mild to moderate hirsutism, with a considerable number of individuals scoring two and three. Conversely, the back and buttocks showed predominantly minimal to no hirsutism, with the majority of participants scoring 0 and 1. There was no discernible difference in mean mFG scores between age groups, according to statistical analysis (p=0.195). Intriguingly, rates of hirsutism were found to be higher in urban versus rural populations, at 78.7% versus 36.6%, respectively (p<0.01). In addition, 80.3% of people who had a positive family history of hirsutism had a mFG score of 8 or higher.

**Conclusion:**

The prevalence of Hirsutism among the normal healthy female population based on the mFG score was relatively high.

## INTRODUCTION

Hirsutism, a condition characterized by excessive hair growth in women, affects up to 10% of women worldwide.^1^ This condition can significantly impact a woman’s quality of life, leading to emotional distress and decreased self-esteem.^2^,^3^ Hirsutism has traditionally been regarded as an indicator of elevated androgen (such as testosterone) levels in females, stemming from increased androgen production either by the adrenal glands or due to ovarian disorders.^4^,^5^ Ovarian causes of hyperandrogenism encompass conditions like polycystic ovarian syndrome (PCOS) and ovarian tumors. Adrenal causes encompass conditions such as Cushing’s syndrome, androgen-producing tumors, and congenital adrenal hyperplasia (CAH), with the latter primarily attributed to 21-hydroxylase deficiency. Less common contributors include the hyperandrogenic-insulin resistant-acanthosis nigricans syndrome (HAIRAN). Additionally, hyperprolactinemia can lead to hirsutism by elevating adrenal dihydroepiandrosterone sulfate (DHEA-S) production. Notably, androgenic medications can also play a pivotal role in hirsutism development.^6^

Interestingly, approximately 20% of patients may present with idiopathic hirsutism (IH), characterized by normal androgen levels and ovarian function. In these cases, it is believed that the increased hair growth may be linked to disruptions in peripheral androgen activity.^7^ IH typically begins shortly after puberty and progresses slowly. PCOS and IH together account for the majority (90%) of hirsutism cases in women. Furthermore, some premenopausal women may experience hirsutism, which can persist for several years after menopause due to a decrease in ovarian estrogen production coupled with ongoing androgen production.^8^

In Pakistan, Hirsutism has been reported to affect up to 20-50% of women, with PCOS being the most common underlying cause.^9^,^10^ However, there is a lack of data on the prevalence of Hirsutism among the normal healthy female population in Pakistan, particularly in Peshawar. The assessment of hair growth in women is primarily done through physical examination, with the mFG score being the most commonly used tool.^11^-^14^ This score is based on the evaluation of hair growth in nine body regions, with scores ranging from 0 to four for each region. A score of eight or more is indicative of hirsutism.^13^ The mentioned cut-off value was established using a cohort mainly comprising Caucasian individuals. However, it is currently recognized that terminal hair growth varies significantly across different races, and hence, the value may not be applicable to different ethnic groups.^15^,^16^ Therefore, it is vital to determine the normative ranges distinct to each race to determine whether a woman has excessive hair.^15^-^17^

Several studies have demonstrated the usefulness of the mFG score in assessing Hirsutism among women.^3^,^11^,^12^ However, there is still a need for more research on the application of this score in different normal healthy female population. Therefore, we conducted our research to determine the prevalence of hirsutism using the international cutoff of mFG score for hirsutism and to find mean of mFG score for our normal healthy female population in Peshawar to compare it with internationally set cutoff mFG to label a female as having hirsutism.

## METHODS

This cross-sectional study was conducted for six months from 14^th^ April 2022 to 13^th^ October 2022 at MTI. Hayatabad Medical Complex in Peshawar.

### Inclusion & Exclusion criteria

A total of 448 normal healthy married women aged 20 to 40 visiting OPD, who had a normal and natural history of conception which was taken as an indirect marker for normal hormonal profile as hyperandrogenism due to any cause may affect fertility and natural/normal conception becomes unlikely, were included, while women having problems with conception, and using assisted methods or medication for fertility, having menstrual irregularities or used medication for menstrual regularity, with comorbidities such as hypertension, diabetes, chronic kidney disease, ischemic heart disease, malignancy, disorders of sexual development, diseases of the glands (pituitary, adrenal, and thyroid), those using medicine for the management of Hirsutism or currently on any medication which can cause hirsutism, were excluded.

### Ethical Approval

The ethical approval for the study was obtained from the Research and Ethical Committee (IREB) of MTI. Hayatabad Medical Complex, Peshawar (Ref. No. 643/HEC/B&PSC/2022; Dated: April 13, 2022).

After taking a consent baseline demographic and clinical characteristics, including age, duration of Hirsutism, education, occupation, residence, history of depilatory practices, and family history of hirsutism were obtained using a structured questionnaire. Clinical evidence of acanthosis nigricans, alopecia, acne, and other signs of virilism was examined. The degree of Hirsutism was assessed using visual techniques, as originally suggested by Ferriman and Gallwey. The density of terminal hairs at several body regions (the lip, lower abdomen, thigh, upper arm, chest, upper abdomen, chin, forearm, lower leg, and upper back) was scored on a scale of 0 to 4. The total score was calculated by averaging the results across all domains. A score of 8 or above on the Ferriman-Gallwey test was considered indicative of Hirsutism.

The information gathered was assessed using SPSS version 22.0. Statistical measures such as mean and standard deviation were employed for continuous data, while frequency and percentage were used for categorical data. The mean mFG Score was assessed with respect to age group using an independent sample T-test. To establish the association between categorical variables, a Chi-square test was applied. The impact of residence, family history of Hirsutism, acne, and acanthosis nigricans on mFG score were analyzed and statistical significance was defined as a p-value ≤ 0.05.

## RESULTS

The mean age of the enrolled participants was 28.75± 5.51 years. 85.7% of women had a duration of Hirsutism of <6 years, and 13.2% had it for > 12 years. Around 15.8% of women had a family history of Hirsutism. (Table-I)

**Table-I:** Baseline demographic & clinical characteristics (n=448).

Variables		n(%)
Age (years); Mean±SD		28.75±5.51
Duration of Hirsutism	<6 years	384(85.7)
	6-12 years	5(1.1)
	>12 years	59(13.2)
Age Group	20-30 years	321(71.7)
	31-40 years	127(28.3)
Education	Illiterate	253(56.5)
	Literate	195(43.5)
Occupation	Employed	62(13.8)
	Unemployed	386(86.2)
Residence	Rural	232(51.8)
	Urban	216(48.2)
History of Depilatory practices	Cream	255(56.9)
	Wax	125(27.9)
	Razor	49(10.9)
	Other	19(4.2)
Acne	Yes	78(17.4)
	No	370(82.6)
Acanthosis Nigricans	Yes	22(4.9)
	No	426(95.1)
Family History of Hirsutism	Yes	71(15.8)
	No	377(84.2)

The mean mFG score of the studied population was 8.89 ± 4.33, 255 (56.9%) of the individuals had a mFG score of ≥ 8. These people showed mild hirsutism in 52.0% of cases, moderate hirsutism in 4.5% of cases, and severe hirsutism in 0.4% of cases. The distribution of hair growth scores across various body regions, ranging from Score 0 (no hirsutism) to Score 4 (severe hirsuitism) are specified in Table-II. Among the most significant findings, mild to moderate hirsutism (mFG score 2-3) in the lower abdomen and thigh region was observed in most cases. Conversely, the back and buttocks showed predominantly minimal to no hirsutism, with the majority of participants scoring 0 and 1.

**Table-II:** Modified Ferriman-Gallwey Score by body region.

Body Area	Mean±SD	Score 0	Score 1	Score 2	Score 3	Score 4
Upper Lip	2.35±0.58	107	215	107	14	5
Chin	1.43±0.70	263	140	33	8	4
Chest	1.30±0.65	149	271	24	4	-
Upper Abdomen	1.10±0.83	215	189	38	6	-
Lower Abdomen	0.74±0.59	3	11	268	160	6
Thighs	0.63±0.69	29	224	171	22	2
Back	0.55±0.78	293	136	15	4	-
Arms	0.42±0.66	31	270	131	15	1
Buttocks	0.40±0.60	296	127	15	10	-

No statistically significant difference was observed in the mean mFG scores with respect to age group (p=0.195). However, the mean mFG of the younger age group (9.05 ± 4.28) is higher than that of the older age group (8.46 ± 4.43).

The urban population has higher prevalence of hirsutism as compared to rural i.e. 78.7% vs. 36.6% (p<0.01). Moreover, 80.3% of those with positive family history of hirsutism showed mFG score ≥ 8 (Table-III). No significant difference was observed in the rural and urban areas concerning the depilatory practices.

**Table-III:** Participant characteristics with respect to Modified Ferriman-Gallwey Score.

Variables		mFG score		p-value

		0-7, n(%)	≥ 8, n(%)	
Residence	Urban	46(21.3)	170(78.7)	0.000*
	Rural	147(63.4)	85(36.6)	
Family History of Hirsutism	Yes	14(19.7)	57(80.3)	0.005*
	No	179(47.5)	198(52.5)	
Acne	Present	14(7.3)	64(25.1)	0.001*
	Absent	179(92.7)	191(74.9)	
Acanthosis Nigricans	Present	4(2.1)	18(7.1)	0.283
	Absent	189(97.9)	237(92.9)	

## DISCUSSION

In this study, we conducted a comprehensive assessment of hirsutism using the modified Ferriman and Gallwey (mFG) scoring system across nine body regions. Our findings revealed that the lower abdomen and thigh region were the body region with most hair growth. The mean mFG score was 8.89 ± 4.33, with a significant proportion (56.9%) of participants scoring ≥ 8, indicating the presence of hirsutism. This contrasts with previous Indian studies, which reported lower mean mFG scores of 5.5^18^ and higher scores of 12.44 ± 3.64 and 13.5 ± 4.6.^19^,^20^ These variations could be attributed to differences in study populations. To define hirsutism in our study, we employed a standardized criterion that required the presence of terminal hair in at least one androgen-dependent site. Our results suggest the need for a higher cutoff value than 8 for diagnosing hirsutism in the local population.

While our patients had a mean age of 28.75 ± 5.51 years, which was higher than some previous studies,^21^,^22^ the most common age range was 20 to 30 years. Interestingly, the mean mFG score was higher in the younger age group than in the older age group. This differs from some prior findings, such as DeUgarte et al., who found no correlation between age and mFG score. Conversely, Sagsoz and colleagues reported a negative correlation between age and total mFG score.^23^,^24^ Lumezi et al. noted an increase in mFG scores with age, with a mean score of 2.4 for all women.^25^ The duration of hirsutism in our patients (1 to 5 years) aligned with some studies but diverged from others.^19^,^21^,^22^,^26^ Abut 15.8% of our patients had a positive family history of hirsuitism, falling within the reported range of 16% to 45% in other studies.^19^,^22^,^26^,^27^

Among the variables studied, residence, family history of hirsutism and acne, showed a significant association with the mFG score in our study. In contrast, other studies have found significant associations between hirsutism and conditions like metabolic syndrome and polycystic ovary syndrome (PCOS).^20^ Notably, elevated calculated free testosterone (FT) levels were significantly associated with a total mFG score of ≥ 7, suggesting a potential link between hirsutism and hyperandrogenic disorders. While there is a weak correlation between hirsutism scores and serum androgen levels, higher serum FT levels are commonly observed in women with hirsutism, indicating the presence of underlying hyperandrogenism. Further investigation is warranted to determine the specific etiology in these cases.^8^

### Limitations

It includes the relatively small sample size which may restrict the generalizability of our findings to other populations. Second, our study was conducted at a single center and thus may not be representative of the entire population of Peshawar. Furthermore, a cross-sectional design can only establish an association between Hirsutism and other variables but cannot establish causality or temporal relationships between them. Future studies with larger sample sizes and a multicenter design are needed to further investigate the prevalence and clinical implications of Hirsutism in this population.

## CONCLUSION

Hirsuitism as indicated by the mFG score among the normal healthy female population was relatively high. Lower abdomen and thigh region were the regions denoted with mild to moderate hirsutism by most participants. We suggest that the mFG score is useful for evaluating Hirsutism in this population. Further research is needed to establish a standardized cut-off for this score as our normal healthy female has slightly higher mean mFG score compare to what we follow internationally to label someone as having hirsutism. Moreover, our results underscore the importance of raising awareness and understanding about Hirsutism and the need to consider potential underlying causes of this condition.

### Authors’ Contribution:

**SB:** Substantial contributions to conception and design, acquisition of data, analysis and interpretation of data.

**SEM:** Participated in analysis, literature review and interpretation of data, and helped in drafting the manuscript.

**SZ:** Participated in data collection, did literature review and interpretation of data.

**JJ:** Helped in data collection, did literature review and interpretation of data.

All authors provided final approval for publication of the manuscript and are responsible for the integrity of the study.
